# Experimental evidence for superionic Fe–C alloy revealed by shear softening in Earth’s inner core

**DOI:** 10.1093/nsr/nwaf419

**Published:** 2025-09-26

**Authors:** Yuqian Huang, Yu He, Youjun Zhang, Jun Li, Long Hao, Bo Gan, Gang Jiang, Qiang Wu, Ho-kwang Mao

**Affiliations:** Institute of Atomic and Molecular Physics, Sichuan University, Chengdu 610065, China; State Key Laboratory of Critical Mineral Research and Exploration, Institute of Geochemistry, Chinese Academy of Sciences, Guiyang 550081, China; Key Laboratory of High-Temperature and High-Pressure Study of the Earth’s Interior, Institute of Geochemistry, Chinese Academy of Sciences, Guiyang 550081, China; Institute of Atomic and Molecular Physics, Sichuan University, Chengdu 610065, China; State Key Laboratory of Intelligent Construction and Healthy Operation and Maintenance of Deep Underground Engineering, Sichuan University, Chengdu 610065, China; National Key Laboratory for Shock Wave and Detonation Physics, Institute of Fluid Physics, China Academy of Engineering Physics, Mianyang 621900, China; National Key Laboratory for Shock Wave and Detonation Physics, Institute of Fluid Physics, China Academy of Engineering Physics, Mianyang 621900, China; Institute of Atomic and Molecular Physics, Sichuan University, Chengdu 610065, China; Institute of Atomic and Molecular Physics, Sichuan University, Chengdu 610065, China; National Key Laboratory for Shock Wave and Detonation Physics, Institute of Fluid Physics, China Academy of Engineering Physics, Mianyang 621900, China; Center for High Pressure Science and Technology Advanced Research, Shanghai 201203, China

**Keywords:** Earth’s core, superionic phase, iron alloys, high pressure and high temperature, Poisson’s ratio

## Abstract

Seismic observations reveal that Earth’s inner core is rigid yet displays ultralow shear wave velocities (*V*_s_) and an ultra-high Poisson’s ratio (∼0.45). While superionic behavior in iron–light element alloys (e.g. carbon) has been proposed to explain these features, experimental validation has been lacking. Here, we combine dynamic high pressure–temperature (*P-T*) experiments with *ab initio* molecular dynamics simulations to investigate the elastic properties and atomic behaviors of hexagonal close-packed (hcp) Fe–1.5 wt% C (Fe-1.5C) alloy under core-like conditions. Theoretical simulations reveal a temperature-induced transition to a superionic state in the hcp-Fe–1.5C alloy above *T/T*_m_ ≈ 0.68 (*T*_m_: melting temperature) under high-pressure conditions. This transition provides a physical explanation for both the ∼23% reduction in *V*_s_ relative to pure Fe and the elevated Poisson’s ratio (∼0.43) measured for the Fe–1.5C alloy at 140 GPa and *T/T*_m_ ≈ 0.83 in our shock-wave experiments. Our findings suggest that the inner core may have liquid-like softness arising from both the superionic diffusion of light elements and the atomic collective motion of iron atoms. This work experimentally confirms a previously unrecognized state of matter in the inner core, reconciling long-standing geophysical and seismological discrepancies.

## Teaser

Superionic behavior in iron-carbon alloys at core conditions provides a key mechanism for inner-core shear softening.

## INTRODUCTION

Seismological observations have revealed a solid yet soft inner core at Earth’s deepest interior, encased within a molten outer core [1,2]. The inner core is mainly composed of iron (Fe) but exhibits a 3%–5% density deficit relative to pure iron [3], suggesting the incorporation of some light elements, such as Si, O, C, S and H [4]. During the growth of the inner core from the outer core, latent heat is released and light elements preferentially partition, providing a critical energy source for core convection [4], which drives Earth’s magnetic field. Despite the inner core’s fundamental role in the geodynamo, our understanding of its thermodynamic properties and the origin of its anomalous seismic characteristics is still rather limited. Of particular note is that its observed shear wave velocity (*V*_s_) ranges from 3.4 to 3.6 km/s [1]—significantly lower than values predicted by using mineralogical experiments and calculations from iron at Earth’s inner-core pressure [5,6]. Consequently, the inner core exhibits an ultra-high Poisson’s ratio, constrained to 0.44–0.45—comparable to that of rubber and the softest metals [7]. Unraveling the genesis of these remarkable seismic properties holds great significance for our comprehension of the structure, composition and dynamics of the inner core.

Several hypotheses have been proposed from recent experimental and theoretical investigations to explain the enigmatic properties of Earth’s inner core [8,9]. Theoretical calculations on elasticity suggest that the inner core, characterized by its pronounced softness, may consist of an iron with a body-centered cubic (bcc) lattice [10]. First-principles simulations have shown a substantial shear softening in bcc-Fe, which could elevate the Poisson’s ratio to values that are consistent with seismological observations [11]. This low shear modulus in bcc-Fe has been attributed to the temperature-induced self-diffusion of iron atoms in theory [12–14]. Although bcc-phase iron may achieve mechanical stabilization via self-diffusion and thermodynamic stabilization through doping with light elements [15], direct experimental evidence remains limited. Conversely, recent high *P-T* experiments using *in situ* X-ray absorption spectroscopy [16] and X-ray diffraction [17,18] have verified the stability of hexagonal close-packed (hcp)-Fe up to its melting point. Shock-compression experiments and machine-learning-enhanced simulations of hcp-Fe reveal that collective atomic motion near premelting conditions (*T/T*_m_ > 0.96) leads to a dramatic reduction in *V*_s_ and a significant increase in the Poisson’s ratio [19]. These findings provide a plausible explanation for the observed seismic softening of the inner core. Nevertheless, these hypotheses are primarily developed within the framework of a pure iron lattice. Light elements have been shown to exert profound effects on the crystal structure and elastic properties of iron [20,21]. Their inclusion is crucial for advancing current models and offering a more comprehensive explanation for the anomalous properties of the inner core.

Carbon has been proposed to be an indispensable component in Earth’s core due to its high abundance in chondrites [22] and strong affinity for iron [23], influencing the unique physical properties of Earth's core [24]. High-pressure experiments and theoretical investigations reveal that iron carbide, particularly Fe_7_C_3_, exhibits a high Poisson’s ratio and low *V*_s_, which are overall consistent with seismic observations [25]. These pioneering works underscore the potential role of carbon in explaining the anomalous elastic properties of the inner core, as supported by some *ab initio* molecular dynamics (AIMD) calculations [26]. Unfortunately, none of these examined iron carbides can simultaneously reconcile both the density and sound-wave velocities of the inner core [27]. Theoretical studies on the phase relation of Fe–Fe_7_C_3_ further suggest that Fe_7_C_3_ is unlikely to be a dominant phase in the inner core [28]. Alternatively, interstitial carbon doped in hcp-Fe has been predicted to lower the *V*_s_ of iron while enhancing its seismic anisotropy under high pressure [29]. Under certain *P*–*T* conditions, interstitial carbon in hcp-Fe is predicted to transition into a superionic state [30], leading to an exceptionally low *V*_s_ and a high Poisson’s ratio of 0.43. Comparable superionic behaviors have also been proposed for interstitial hydrogen (H)- and oxygen (O)-bearing hcp-Fe [30,31]. However, the synthesis of homogeneous Fe alloys containing interstitial H or O remains highly challenging, as both elements exhibit extremely low solubility in solid iron and are difficult to stabilize or recover under ambient conditions [32,33]. By contrast, carbon displays significantly higher solubility in solid iron, which enables the preparation of homogeneous, well-defined solid-solution alloys. Therefore, Fe–C solid solutions can serve as representative systems for experimentally testing theoretical predictions of superionic behavior under Earth’s core conditions.

In this study, we conducted high *P*–*T* experiments via shock compression and AIMD simulations to examine the influence of interstitial carbon on the thermodynamic behavior and elastic properties of solid iron under conditions relevant to those at Earth’s core. Our experimental results reveal a pronounced softening of the *V*_s_, accompanied by a significant increase in the Poisson’s ratio, in hcp-Fe alloyed with 1.5 wt% interstitial carbon under Earth’s solid-core-like conditions. Our theoretical simulations suggest that this shear softening is a consequence of extensive carbon atomic diffusion in Fe–C alloys within the superionic state under the corresponding *P*–*T* conditions. Combined with the observed collective atomic motion in premelting hcp-Fe [19], our findings imply that both the superionic diffusion of light elements, such as carbon, and the collective atomic motion in solid iron under inner-core conditions jointly contribute to the strong shear softening and the ultra-high Poisson’s ratio.

## RESULTS AND DISCUSSION

### Sound velocities of Fe–C alloy at high *P*–*T* along the Hugoniot

**Figure 1.**
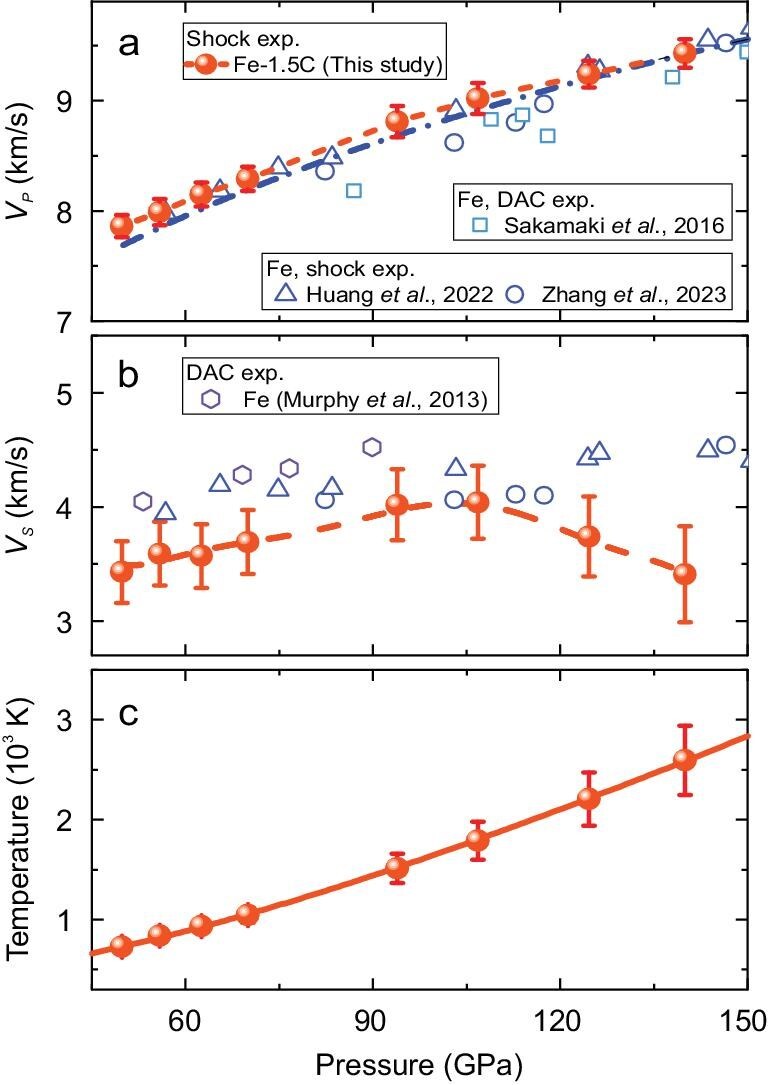
Sound-velocity measurements of Fe-1.5C alloy under high *P*–*T* conditions during shock compression. (a) Measured longitudinal sound velocity of Fe-1.5C alloy as a function of pressure along the Hugoniot. (b) Pressure dependence of shear sound velocity of Fe-1.5C alloy along the Hugoniot, showing a significant drop in shear sound velocity between 105 and 140 GPa. (c) Shock temperature of Fe-1.5C alloy as a function of pressure along the Hugoniot. Solid circles represent results from this study, while open triangles and circles represent shock experiments on pure iron from Huang *et al.* (2022) [20] and Zhang *et al.* (2023) [19], respectively. Squares indicate experimental results on pure iron in static diamond anvil cell (DAC) compression at high pressure and 2300–3000 K by Sakamaki *et al.* (2016) [48]. The dash-dot line shows the fit for the longitudinal sound velocity of pure iron from Huang *et al.* (2022) [20]. Hexagons correspond to static DAC compression experiments on pure iron at ambient temperature by Murphy *et al.* (2013) [49]. The Hugoniot temperatures of Fe-1.5C are calculated by using the measured Hugoniot equation of state.

To investigate the sound-velocity properties of Fe–C alloy under Earth’s core conditions, we synthesized homogeneous Fe–1.5 wt% C (Fe–1.5C) solid-solution alloys by using multi-anvil press apparatus (refer to the Supplementary data for more details). Shock-compression experiments were performed by using two-stage light gas guns at Sichuan University and the Institute of Fluid Physics, China. The reverse-impact technique achieved maximum projectile velocities of >7 km/s, generating shock pressures of 49.8–140.0 GPa with corresponding temperatures of 729–2594 K along the Hugoniot (Fig. 1c and Fig. S1). Photon Doppler velocimetry [34] measurements provided sub-nanosecond temporal resolution particle velocity measurements, enabling precise recording of the wave profiles at the sample/LiF interface (Fig. S1b). Compressional sound velocities (*V*_p_) were determined from the time interval between the arrival of the shock wave and the rarefaction wave at the sample/LiF interface, with uncertainties of <2% (Table S1). Bulk wave velocities (*V*_b_) were obtained from the time of elastic–plastic transition (Fig. S2), allowing determination of the *V*_s_ and Poisson’s ratio through established relationships (see ‘Methods’ for details).

Our experiments reveal distinct behaviors in the *V*_p_ and *V*_s_ wave velocities of the Fe–1.5C alloy along the Hugoniot pressure (Fig. 1a and b). The *V*_p_ of the Fe–1.5C alloy is ∼1%–2% faster than that of pure iron between 50 and 140 GPa along the Hugoniot (Fig. 1a). Notably, the quasi-linear *V*_p_–density correlation (9.24–9.43 km/s at 10.97–11.12 g/cm³) demonstrates a systematic 2%–3% enhancement in *V*_p_ relative to hcp-Fe under equivalent conditions (Fig. S3a), indicating subtle but measurable effects of interstitial carbon on the compressional wave characteristics of the iron. In contrast, the *V*_s_ of the Fe–1.5C alloy displays anomalous changes with two distinct regimes. Below ∼107 GPa, 1.5 wt% carbon doping reduces the *V*_s_ of pure iron by 1%–2%. However, above this threshold along the Hugoniot, we observe a dramatic ∼15% decrease in *V*_s_ from 4.0 to 3.4 km/s (Fig. 1b and Fig. S3a), culminating in a 23% reduction compared with pure iron at 140 GPa. This non-linear deviation suggests a transition in the elastic response of the Fe–1.5C alloy that is distinct from that of pure iron. In contrast, the measured Hugoniot equation of state for the Fe–1.5C alloy over the *P*–*T* range of 50–185 GPa and 720–3750 K exhibits continuous compressibility [35], suggesting that the Fe–C solid solution remains stable without decomposition at ≤140 GPa and 3750 K under shock compression. Previous first-principles calculations predicted linear pressure hardening for carbon-doped hcp-Fe at 0 K [29]. Static high *P*–*T* experiments indicate that the sound velocities of iron alloys tend to soften with rising temperature [5,20,36], suggesting competing mechanisms between pressure-induced hardening and temperature-driven softening. In our shock-compression experiments (Fig. 1c), the concurrent increases in pressure and temperature along the Hugoniot demonstrate thermal softening dominance above the critical threshold (∼110 GPa and 1900 K), where *V*_s_ undergoes a marked reduction.

To quantitatively validate the temperature-dependent elastic softening observed in the shock experiments, we performed AIMD simulations on the Fe–1.5C alloy at 140 GPa over a temperature range of 1500–3000 K. Elastic constants (*C*_11_, *C*_12_, *C*_13_, *C*_33_ and *C*_44_) were calculated via stress–strain relations, with bulk and shear moduli, by using the Voigt average method. The calculated sound velocities, as shown in Fig. S4, exhibit significant thermal softening. Specifically, *V*_p_ decreases from 9.7 km/s at 1500 K to 9.0 km/s at 3000 K, while *V*_s_ decreases more markedly from 4.3 to 3.5 km/s, with pronounced softening beginning at ∼2000 K. These computational results reproduce the experimental observation of *V*_s_ reduction above ∼110 GPa, confirming the dominant role of temperature in modulating the shear wave elasticity of the Fe–1.5C alloy under extreme conditions. The convergence of experimental data and AIMD simulations reveals distinct elastic anomalies, underscoring the need for atomic-scale characterization to decipher the underlying crystallographic mechanisms driving these behaviors.

### Atomic origin of shear softening and superionic transition

**Figure 2.**
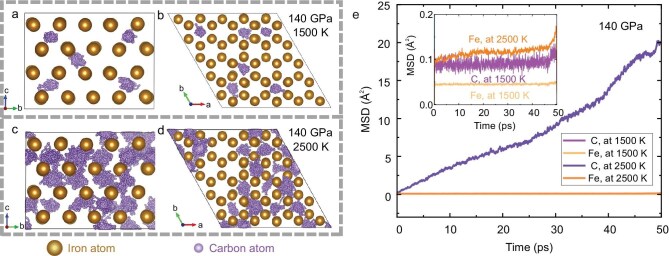
Atomic trajectories and MSD of Fe–1.5C alloy from AIMD simulations at 1500 and 2500 K under 140 GPa. (a–d) Atomic trajectories in a single supercell at 140 GPa and temperatures of (a and b) 1500 K and (c and d) 2500 K, showing motion along the (a and c) *a*-axis and (b and d) *c*-axis, depicting the phase transition from (a and b) solid hcp to (c and d) superionic hcp. Iron and carbon atoms are shown as gold and purple spheres, respectively. (e) Averaged MSDs of iron (light yellow and orange lines) and carbon (light purple and blue lines) atoms as a function of time at 1500 and 2500 K under 140 GPa. The inset in (e) zooms in on the MSDs within the range of 0.0–0.2 Å² over 50 ps. These data provide insight into the dynamic behavior and structural characteristics of the Fe–C system under extreme *P*–*T* conditions.

Elucidating the atomic-scale mechanism of temperature-dependent sound-velocity anomalies in the Fe–1.5C alloy under 140 GPa, we performed AIMD simulations with a timescale spanning 50 ps across multiple temperatures. Prior theoretical calculations reported that hcp-Fe doped with ∼6 at% interstitial carbon could stabilize itself at >9 GPa [37]. Through the quantitative analysis of mean-square displacements (MSDs), we determined temperature-dependent diffusion coefficients for both iron (*D*_Fe_) and carbon (*D*_C_) atoms. The diffusion coefficients of Fe and C elements are usually considered as crucial parameters to delineate three distinct phase regimes: (i) ideal crystalline solid (*D*_Fe_ ≈ 0, *D*_C_ ≈ 0), (ii) superionic state (*D*_Fe_ ≈ 0, *D*_C_ > 0) and (iii) liquid phase (*D*_Fe_ > 0, *D*_C_ > 0) [30]. As visualized in Fig. 2a–d, iron atoms maintain hcp-lattice order, while carbon atoms exhibit dynamic evolution from the initial interstitial sites.

At 1500 K, carbon atoms exhibit localized vibration restricted to their original crystallographic sites within the hcp-iron lattice, with thermal motions confined to the intralayer direction (Fig. 2a and b). The MSDs of both the carbon and the iron atoms within the hcp structure remain nearly constant throughout the simulation time, resulting in the diffusion coefficients of both being nearly zero (Fig. 2e), indicative of a frozen configuration with typical solid-hcp characteristics. However, at 2500 K, the thermal activation significantly intensifies atomic vibrations, leading to striking contrasts. The MSD of iron atoms at 2500 K exhibits relatively larger fluctuations compared with that at 1500 K (Fig. 2e), though not drastically so. In contrast, the MSD and atomic trajectories of carbon atoms show much larger amplitudes, corresponding to a *D*_C_ of 0.4 × 10^–8^ m^2^ s^–1^ (Fig. 2c–e). Notably, the carbon atoms move to neighboring interstitial sites and even traverse multiple interstitial sites among the iron atoms, with their trajectories distributed in a contiguous manner. Additionally, some carbon atoms continuously diffuse along the interlayer direction, parallel to the *c*-axis (Fig. 2c and d). The non-zero diffusion constant of carbon suggests a structural transition of the Fe–1.5C alloy into a superionic-hcp phase, in which the carbon atoms exhibit liquid-like behavior. The *P*–*T* conditions for solid-to-superionic transition, as determined by using AIMD simulations, coincide with the region in which shear softening is observed experimentally and theoretically (Fig. 1b and Fig. S3a).

The distinct behaviors of carbon atoms in the hcp-Fe alloy at 1500 and 2500 K illustrate that temperature acts as a dynamical driving force for the superionic transition. The weak van der Waals interactions between the Fe and C allow carbon mobility below the melting point, while the strong metallic Fe–Fe bonds maintain the lattice framework. Additionally, similar results recently reported for superionic face-centered cubic (fcc)-FeH under high *P*–*T* conditions have also been proposed to be associated with the atomic mass and radius [38]. Lighter atoms such as H and C are expected to move rapidly under identical kinetic energy conditions.

To quantify the diffusion behavior, we further calculated the diffusion coefficients of carbon atoms for the Fe–1.5C alloy across various *P*–*T* conditions, at fixed densities of 11.224, 11.506 and 11.773 g/cm^3^. At lower temperatures, the diffusion coefficients show a negative correlation with pressure, indicating the confinement of interstitial carbon atoms. In contrast, a temperature-induced enhancement in diffusion becomes evident at ∼2000 K (Fig. S5), coinciding with the elastic anomalies discussed above. To further assess the influence of the carbon concentration on superionic behavior, we performed additional AIMD simulations in the canonical ensemble (constant number of particles, volume, and temperature, NVT) at ∼166 GPa and 3000 K for 50 ps. Three compositions were examined at a constant volume of 818.09 Å^3^ (NVT ensemble): Fe–0.6C (Fe_100_C_3_), Fe–1.5C (Fe_100_C_7_) and Fe–1.9C (Fe_100_C_9_) (Fig. S6). The calculated MSDs for the Fe and C atoms yield carbon diffusion coefficients of ∼0.2 × 10^–8^ m^2^ s^–1^ for all three compositions. These results indicate that superionic behavior is preserved in Fe–C solid-solution alloys containing at ≥0.6–1.9 wt% carbon under conditions relevant to Earth’s inner core.

### Phase diagram of Fe–1.5C alloy under Earth’s core conditions

**Figure 3.**
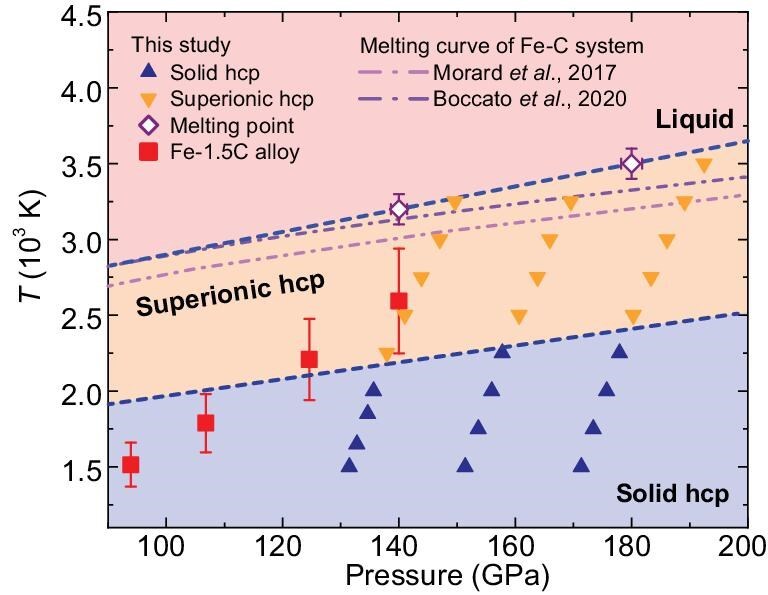
Predicted phase diagram of Fe–C alloy from simulations, with comparisons to the experimental *P*–*T* conditions. Our shock experiments indicate that the Fe-1.5C alloy has experienced a transition from the ideal solid-hcp state to the superionic-hcp state. The upward and downward triangles represent the solid hcp and superionic hcp phases, respectively. Open diamonds denote the calculated melting points of the Fe-1.5C alloy by using the two-phase method. Solid squares show the experimental *P*–*T* conditions of the Fe-1.5C alloy along the Hugoniot. The dotted and dash-dot lines represent the melting curves of the Fe–C systems from Morard *et al.* (2017) [40] and Boccato *et al.* (2020) [39], respectively. The dashed lines refer to the solid-superionic phase boundary and the fitted melting curve for the Fe-1.5C alloy, respectively. The light-blue, light-orange and light-pink shaded regions distinguish the solid hcp, superionic hcp and liquid regions, respectively.

We performed calculations at pressures ranging from 130 to 190 GPa and temperatures between 1500 and 3500 K to map the *P*–*T* phase diagram of Fe–1.5C, including the solid–superionic–liquid phase transitions under relevant core conditions. Notably, both C and Fe exhibit pronounced diffusion in the superionic–liquid coexisting phase, which is distinct from their diffusion behaviors in a normal solid and superionic state (Fig. S7). This characteristic diffusion feature is therefore used to define the superionic–liquid phase boundary. Melting temperatures (*T*_m_) were determined by using the two-phase method, which mitigates superheating effects in simulations. Our results identify three distinct thermodynamic regimes (Fig. 3): solid-hcp phase (carbon atoms localized at interstitial sites), superionic-hcp phase (liquid-like carbon diffusion within a rigid iron lattice) and liquid phase (complete lattice disintegration). The melting curve rises from ∼3200 K at 140 GPa to ∼3500 K under 180 GPa (Fig. 3 and Fig. S8). These findings are in close agreement with *in situ* laser-heated X-ray absorption spectroscopy experiments of Fe–C alloy containing 1.5 wt% C [39], though they slightly exceed predictions for the Fe–Fe_3_C system [40], likely due to differences in carbon incorporation mechanisms within the hcp-Fe lattice. As expected, the temperature for the solid–superionic transition (*T*_t_) increases monotonically with the pressure, maintaining a constant *T*_t_*/T*_m_ ratio of 0.68 under the investigated *P-T* conditions.

**Figure 4.**
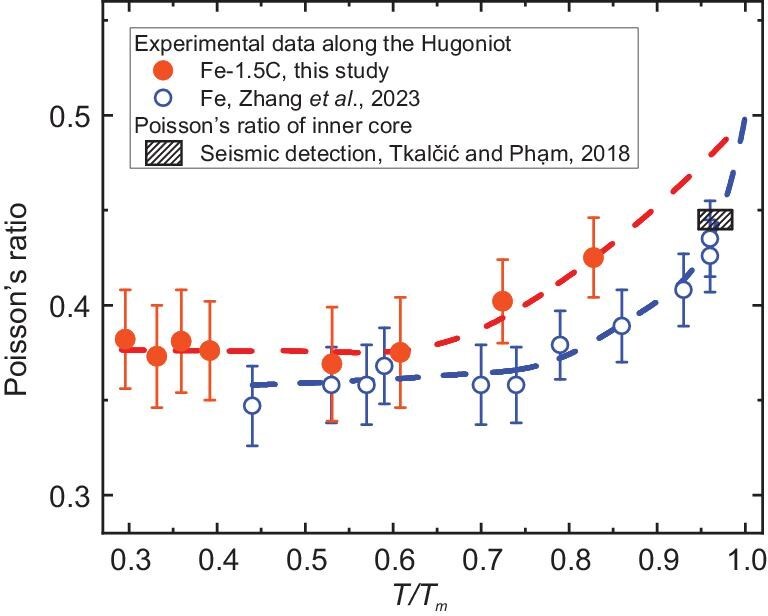
Poisson’s ratio of Fe-1.5C alloy and pure Fe as a function of the ratio of temperature to melting point (*T/T*_m_). Solid circles with error bars represent the experimental data for the Fe-1.5C alloy from this study along the Hugoniot, while open circles with error bars correspond to data for pure iron from Zhang *et al.* (2023) [19]. The striped rectangle indicates the Poisson’s ratio of the inner core as constrained by seismic detections [1]. The observed difference in the Poisson’s ratio between Fe-1.5C and Fe at relatively low *T/T*_m_ may be attributed to the influence of interstitial carbon. As *T/T*_m_ grows to >0.68, the Fe-1.5C alloy transitions from solid hcp to superionic hcp; when *T/T*_m_ surpasses 0.95, collective atomic motions in hcp-Fe are initiated.

Within the pressure range of 90–110 GPa, *T*_H_*/T*_m_ (*T*_H_: shock temperature) falls to between 0.51 and 0.63, indicating that the Fe–1.5C alloy maintains a stable solid-hcp structure, as evidenced by the nearly linear correlation between the sound velocities and pressure/density (Fig. 1a and b, and Fig. S3a). At >110 GPa, the alloy initiates a transition towards the superionic state, marked by a change in the slope of *V*_s_ with further increases in the shock pressure. The complete phase transformation is achieved at 117 GPa and ∼2050 K, with *T*_H_*/T*_m_ increasing to 0.68. Our shock Hugoniot crosses the solid–superionic boundary, with experimental conditions ranging from 124.6 to 140.0 GPa and corresponding *T*_H_*/T*_m_ values of ∼0.72–0.81, which are firmly within the superionic regime (Fig. 3). This phase transition is accompanied by significantly elastic anomalies, inducing a 15% reduction in *V*_s_ (from 4.0 to 3.4 km/s) and a 13% increase in the Poisson’s ratio (from 0.38 to 0.43). Remarkably, these experimental values align closely with those of seismological observations of the inner core, where *V*_s_ ranges from 3.4 to 3.6 km/s and the Poisson’s ratio falls between 0.44 and 0.45 at *T*_H_*/T*_m_ of 0.95–0.99 [41] (Fig. 4). The agreement between our experimental measurements and geophysical data establishes superionic carbon diffusion as the mechanism that reconciles the ‘inner-core paradox’: the simultaneous rigidity for seismic wave propagation and fluid-like elasticity.

### Poisson’s ratio of Fe–C alloy under Earth’s core conditions

The Poisson’s ratio of the Fe–1.5C alloy at high *P*–*T* is derived from our measured *V*_p_ and *V*_s_ values along the Hugoniot (Fig. 4 and Fig. S3b). Quantitative analysis of the Poisson’s ratio evolution reveals two distinct regimes: (i) below a *T/T*_m_ value of 0.68, the Poisson’s ratio remains nearly constant at 0.38 with minimal variation in the solid-hcp phase behavior; (ii) above a *T/T*_m_ value of ∼0.68, the Poisson’s ratio converges toward inner-core values, indicating complete superionic transition, with carbon atoms freely diffusing within the hcp lattice. These findings demonstrate that even a small fraction of superionic carbon can significantly elevate the Poisson’s ratio to match the anomalously high values observed in the inner core.

Along the adiabatic thermal profile of the inner core, where *T/T*_m_ is estimated to 0.96–0.99 (Fig. 4), in addition to the collective motion of pure iron [19], superionic carbon also freely diffuses in the premelting hcp lattice. Our experimental results on the Fe–1.5C alloy provide compelling evidence that hcp-Fe alloyed with superionic light elements, such as carbon, can reproduce seismic characteristics that are strikingly similar to those of the inner core, including ultralow *V*_s_ and an ultra-high Poisson’s ratio. These findings are consistent with previous theoretical predictions of superionicity-induced elastic softening [30,31,38]. Extending this framework, further investigations into the potential superionic behaviors of interstitial H- and O-bearing iron alloys under inner-core conditions are essential for developing a more comprehensive understanding of the shear properties of the inner core.

### Implications of superionic Fe–C alloy in Earth’s solid inner core

Earth’s inner core is predominantly composed of an iron–nickel alloy with trace amounts of light elements. Any viable compositional model must reconcile with seismic observations. Integrating our findings with prior studies, we propose that the softness of the inner core can be attributed to the intriguing atomic diffusion within Fe–C alloys. Zhang *et al.* [19] recently demonstrated that the collective motion of iron atoms occurs spontaneously in premelting hcp-Fe along the inner-core adiabat, yielding an ultra-high Poisson’s ratio of 0.43–0.44. When coupled with the diffusive behavior of carbon atoms within the hcp lattice, the Poisson’s ratio of the superionic Fe–C alloy would much more closely match that of the inner core (0.44–0.45), providing an integrated mechanism that reconciles the observed reduction in *V*_s_ and increase in the Poisson’s ratio (Fig. 4). Nonetheless, further investigations of the Fe–C alloy through higher *P*–*T* experiments would provide critical constraints on the evaluation of the relative importance of these two effects.

**Figure 5.**
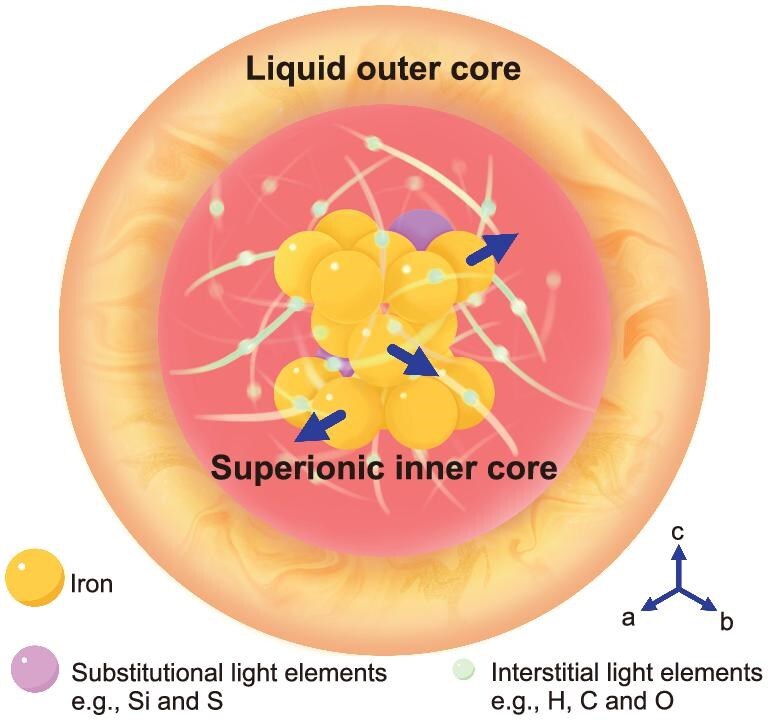
Schematic of atomic diffusion in superionic iron alloys under conditions relevant to the inner core. The iron atoms with a hcp lattice under premelting conditions diffusively migrate along [100] and [010] crystallographic directions. Substitutional light elements (e.g., Si, S) occupy lattice sites, while interstitial light elements (e.g., H, C, O) diffuse freely within the lattice.

Importantly, our analysis of the Hugoniot equation of state for the Fe–1.5C alloy [35] reveals no significant density changes associated with the transition of solid hcp to superionic hcp at >124.6 GPa. This suggests that the superionic transition has a negligible impact on the upper-bound estimate of carbon content (2.2 wt% [35]) in the inner core, derived from the density deficit. Our proposed mechanism offers an alternative explanation for the Fe₇C₃-composed inner-core hypothesis, which has been challenged due to its inability to fully account for the density deficit. In line with the predictions of He *et al.* [30], we conclude that the inner core may consist of solid iron with liquid-like interstitial light elements (Fig. 5), facilitating ongoing convective and dynamic activities.

These findings have profound implications for understanding the state and dynamics of the core, particularly in relation to the inner-core seismic anisotropy and driving mechanisms of the geodynamo. It is known that the inner-core anisotropy may arise from preferred atomic diffusion pathways [30]. First-principles calculations indicate that interstitial carbon in hcp-lattice Fe can significantly enhance seismic anisotropy [29]. Similarly to the superionic Fe–H alloy [42], the directional motions of both iron and interstitial light elements along energetically favorable pathways could potentially explain the observed anisotropic structure of the inner core. Furthermore, the mobility of atoms in the superionic iron alloy enables chemical convection without bulk melting in the inner core, potentially altering the heat-transfer efficiency in core dynamics and providing an alternative energy source for sustaining the geodynamo. Additionally, the superionic iron alloy can exhibit both electronic and ionic conductivity [30], suggesting that our understanding of the inner core’s electro–thermal transport properties may need refinement. This implies that thermal and/or chemical convection in the inner core may be more complex than previously assumed.

Our combined high *P*–*T* experiments and theoretical simulations regarding the effects of interstitial carbon on pure iron has demonstrated that transition into superionic carbon occurs at *T/T*_m_ above ∼0.68 at core pressures, resulting in dramatic shear softening and an enhanced Poisson’s ratio. These findings provide a robust mechanism for the anomalous seismic characteristics of the inner core, driven by the interplay of collective atomic motions in hcp-Fe and superionic carbon diffusion. The collectively moving iron and liquid-like carbon suggest that the solid inner core may be in a dynamic equilibrium state, with elements undergoing redistribution. Geophysical modeling that takes into account the atomic diffusion behavior is required to understand the impact of atomic diffusion on more geodynamic processes, such as the growth of the inner core. We also noted that there is a density contrast at the inner-core boundary, which has been attributed to the uneven distribution of light elements between the inner and outer core [33,43]. Therefore, further high *P*–*T* mineral physical research is needed to investigate more physical properties of iron doped with superionic light elements.

## METHODS

### Sound-velocity measurements of Fe–C alloy at high pressure and temperature by shock compression

As shown in Fig. S1a, the sample was accelerated to impact the [100]-oriented LiF window [44] at velocities ranging from 3.4 to 7.0 km/s. Upon impact, a shock wave is generated in both the sample and the LiF window (Fig. S1b). The forward shock-wave front arrives at *t*_0_, causing an abrupt jump in the interface particle velocity, which then remains constant. Upon reaching the sample’s rear surface, the backward shock wave reflects, forming a reverse rarefaction sound wave. Then, the pressure is released. The particle velocity drops significantly when the rarefaction sound wave reaches the interface at *t*_1_. Based on the measured time interval (*t*_1_–*t*_0_) and the thickness of the sample (*h*_s_), the Lagrangian longitudinal sound velocity *V*_p_^L^ can be determined from the following equation [45]:


(1) \begin{eqnarray*}
V_{\mathrm{p}}^{\mathrm{L}} = \frac{{{h}_{\mathrm{s}}}}{{\left( {{t}_1 - {t}_0} \right) - {h}_{\mathrm{s}}/{U}_{\mathrm{s}}}}.
\end{eqnarray*}

Then, the longitudinal sound velocity *V*_p_ in the Eulerian coordinates are ascertained from Equation (2):


(2) \begin{eqnarray*}
{V}_{\mathrm{p}} = \frac{{{\rho }_0}}{\rho }V_{\mathrm{p}}^{\mathrm{L}} = \frac{{{\rho }_0}}{\rho }\frac{{{h}_{\mathrm{s}}}}{{\left( {{t}_1 - {t}_0} \right) - {h}_{\mathrm{s}}/{U}_{\mathrm{s}}}},
\end{eqnarray*}

where *ρ*_0_ and *ρ* denote the initial and shocked densities of the sample, respectively; *U*_S_ refers to the shock-wave velocity of the sample under compression.

Once the elastic–plastic transition at *t*_2_ is identified (Fig. S1b), we can deduce the Lagrangian bulk sound velocity (*V*_b_^L^) along the Hugoniot (Fig. S2). By applying a coordinate transformation, we determine the Eulerian bulk sound velocity. Subsequently, the shear sound velocity *V*_s_ and Poisson’s ratio ν can be derived from the following relations:


(3) \begin{eqnarray*}
V_{\mathrm{s}}^2 = \frac{3}{4}\left( {V_{\mathrm{p}}^2 - V_{\mathrm{b}}^2} \right),
\end{eqnarray*}

(4) \begin{eqnarray*}
\nu = \frac{{V_{\mathrm{p}}^2 - 2V_{\mathrm{s}}^2}}{{2\left( {V_{\mathrm{p}}^2 - V_{\mathrm{s}}^2} \right)}}.
\end{eqnarray*}

Uncertainties in the measured sound velocities at the Hugoniot state are estimated by using uncertainty propagation [19]. The direct measurements of the shock and rarefaction fronts in the Fe–1.5C alloy allow a substantial enhancement in the precision of the longitudinal sound velocity and our experiments allow direct estimation of the temperature effect on the sound velocities under high pressure.

### AIMD simulations and model construction

Spin-polarized AIMD simulations were conducted by using the Vienna Ab Initio Simulation Package [46]. Atomic potentials were generated by using the projector augmented-wave method within the generalized gradient approximation framework [47]. The wave functions were represented by plane waves with a cut-off energy of 600 eV. The Fe (3d^6^4s^2^) and C (3s^2^3p^2^) orbitals were treated as valence states. For k-point sampling, only the gamma point was used. A large supercell consisting of 100 Fe atoms and 7 C atoms (Fe–1.5 wt% C) was employed for the AIMD simulations under the conditions of ∼130–190 GPa and 1500–3500 K. The C atoms were randomly distributed at interstitial sites within the hcp lattice. The simulations were performed in the NVT ensemble with a time step of 1 femtosecond (fs), covering a total duration of 50 ps. The diffusion coefficient of ionic carbon was calculated, along with the MSD of the ionic positions (Fig. S5):


(5) \begin{eqnarray*}
\left\langle {{{\left[ {\mathop r\limits^{\rightarrow}(t)} \right]}}^2} \right\rangle = \frac{1}{N}\mathop \sum \limits_{i = 1}^N \left\langle {{{\left[ {\mathop {{r}_i}\limits^{\rightarrow} ( {t + {t}_0} ) - \mathop {{r}_i}\limits^{\rightarrow} ( {{t}_0})} \right]}}^2} \right\rangle ,
\end{eqnarray*}

where $\mathop {{r}_i}\limits^{\rightarrow} ( {{t}})$ is the displacement of the *i*-th ion at time *t* and *N* is the total number of specific ions in the system.

To assess the finite-sized effects in our AIMD calculations, we performed an additional NVT simulation by using an enlarged supercell containing 200 Fe and 14 C atoms at ∼161 GPa and 2500 K for 20 ps. The resulting MSDs are consistent with those obtained from our primary 107-atom model (100 Fe and 7 carbon atoms), indicating that the supercell dimensions have a negligible influence on the simulation outcomes.

## Supplementary Material

nwaf419_Supplemental_File

## Data Availability

All data in the paper are presented in the article and/or Supplementary Data.
