# Supplementary material for: Experimental evidence for superionic Fe–C alloy revealed by shear softening in Earth’s inner core
Source: Natl Sci Rev. 2025 Sep 26;12(11):nwaf419. doi: 10.1093/nsr/nwaf419 (PMC12598642; doi:10.1093/nsr/nwaf419)
Supplement: nwaf419_Supplemental_File [file nwaf419_supplemental_file.docx]

**Supplementary Information for**

**Experimental Evidence for Superionic Fe-C Alloy Revealed by Shear Softening in Earth’s Inner Core**

Yuqian Huang^1,^ ^†^, Yu He^2,3, †^, Youjun Zhang^1, 6, 7*^ Jun Li^4^, Long Hao^4^, Bo Gan^1^, Gang Jiang^1^, Qiang Wu^4^, and Ho-kwang Mao^5^

^1^Institute of Atomic and Molecular Physics, Sichuan University, Chengdu 610065, China.

^2^State Key Laboratory for Critical Mineral Research and Exploration, Institute of Geochemistry, Chinese Academy of Sciences, Guiyang 550081, China.

^3^Key Laboratory of High-Temperature and High-Pressure Study of the Earth’s Interior, Institute of Geochemistry, Chinese Academy of Sciences, Guiyang 550081, China.

^4^National Key Laboratory for Shock Wave and Detonation Physics, Institute of Fluid Physics, China Academy of Engineering Physics, Mianyang 621900, China.

^5^Center for High Pressure Science and Technology Advanced Research, Shanghai 201203, China.

^6^State Key Laboratory of Intelligent Construction and Healthy Operation and Maintenance

of Deep Underground Engineering, Sichuan University, Chengdu 610065, Sichuan, China

^7^Key Laboratory of High Energy Density Physics and Technology of Ministry of Education, Sichuan University, Chengdu 610065, China

**EXPERIMENTAL SECTION**

**Starting materials****.** In the present shock-compression experiments, the Fe-1.5wt.%C were used to be the starting material, same as that has been used in our previous shock studies [1]. The composition of 1.5 wt.% C adopted in this study (1) represents a value within the geophysically plausible range for the inner core [1,2], and (2) corresponds to a concentration that can be incorporated into iron as a stable solid solution at conditions of synthesis, and subsequently quenched to ambient conditions to form a homogeneous starting material. Chemically homogeneous Fe-1.5 wt.% C samples were synthesized from a powder mixture of reagent‐grade iron and graphite, with a mass ratio fixed to 98.5:1.5. The high P-T sintering was conducted at 5-6 GPa and 1800 K in a cubic large-volume press at Sichuan University. The well-sintered samples are cylindrical and ground down to 1.3-1.7 mm in thickness with a diameter of ~10 mm. Each sample was then finely double-polished for the measurements of initial density and sample characterizations at ambient conditions. The resulted average initial density is 7.770 (0.014) g/cm^3^ by using the Archimedean method. Compositional characterizations and phase identification of experimental samples can be found in our previous work [1]. According to X-ray diffraction patterns, the sample contains a major phase of tetragonal-martensite (I4/mmm) and minor phase of cubic-austenite (Fm-3m), both of which are iron-carbon solid solution alloys. Electron probe microanalysis indicates uniform distribution of carbon and iron with an average carbon content of 1.43 (0.26) wt.% or 6.34 (1.20) at%.

**Melting Point prediction by the two-phase method.** The melting temperature was estimated by the two-phase coexistence method. AIMD simulations were performed on a solid-liquid coexistence model in the isothermal-isobaric ensemble (*NPT*), where the number of atoms, pressure, and temperature were held constant. The coexistence model consisted of 428 atoms for Fe_400_C_28_. The time step was set at 1 fs with a total simulation of over 50 ps. The two-phase coexisting states are maintained during our simulation (Fig. S8 and Fig. S9).

**Calculations of Elastic Properties for Fe-C alloy at high P-T.** The elastic properties were determined using AIMD method, which has been extensively employed in our prior investigations [3,4]. The equilibrium volume and unit cell parameters at various temperatures were obtained by performing a series of simulations in the canonical ensemble (NVT) across a grid of volumes and temperatures. For each equilibrium structure, a simulation spanning 20,000 time steps (20 ps) was conducted to ensure the stress field remained hydrostatic. The elastic constants *C_ij_* were computed by applying distortions to the equilibrium structure and solving the resulting stress-strain relationships. We calculated the isothermal bulk modulus (*B_T_*) and shear modulus (*G*) using Voigt average scheme. The adiabatic bulk modulus (*B_S_*) was calculated using the following relationship:

$B_{S}=B_{T}\left( 1+\alpha\gamma T \right)$, (6)

where α is the volumetric thermal expansion coefficient, and γ is the Grüneisen parameter. Based on previous calculation for iron alloys under Earth’s core conditions [5,6], α=1 ×10^−5^/K, and γ=1.5. The adiabatic compressional wave velocity (*V_P_*) and shear wave velocity (*V_S_*) were calculated from the elastic moduli and the density (*ρ*):

$V_{P}=\sqrt{\frac{B+\frac{4G}{3}}{\rho}}$, (7)

$V_{S}=\sqrt{\frac{G}{\rho}}$. (8)

The calculated results are consistent with experimental results as shown in Fig. S4

**FIGURES and TABLES.**


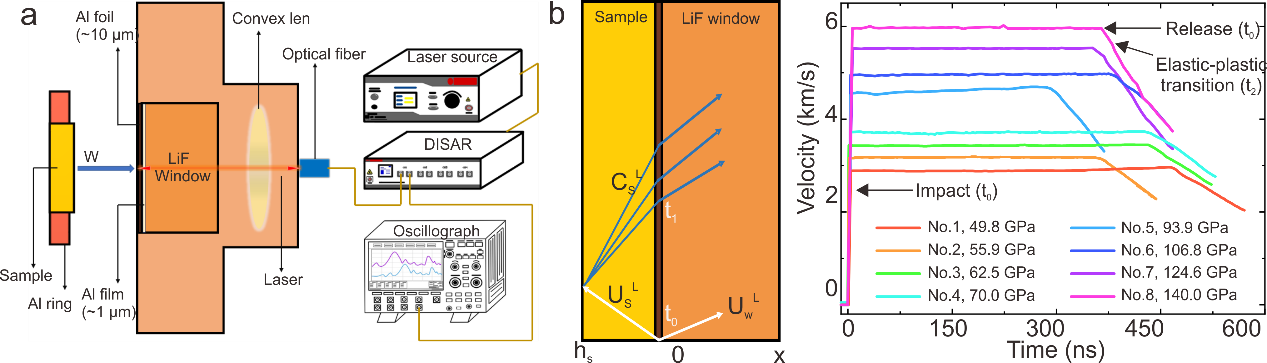


Fig. S1 Schematic of the experimental setup for the high pressure-temperature sound velocity measurements of Fe-1.5wt.%C alloy under shock compression. a Schematic diagram of the reverse-impact experiments for the longitudinal sound velocities of Fe-1.5wt.%C alloy, used as the flyer. The [001] single-crystal lithium fluoride (LiF) target, vapor-deposited with a 1-um aluminum (Al) film, was applied as the transparent window. To avoid noise signals, a 10-μm-thick aluminum foil was typically coated on the impact surface of Al film. Upon impact, the interface particle velocity profiles can be recorded by the Photon Doppler velocimetry (PDV). b Schematic diagram of the wave propagation and the corresponding particle velocity profiles at the sample/LiF window interface, as measured in eight experiments.


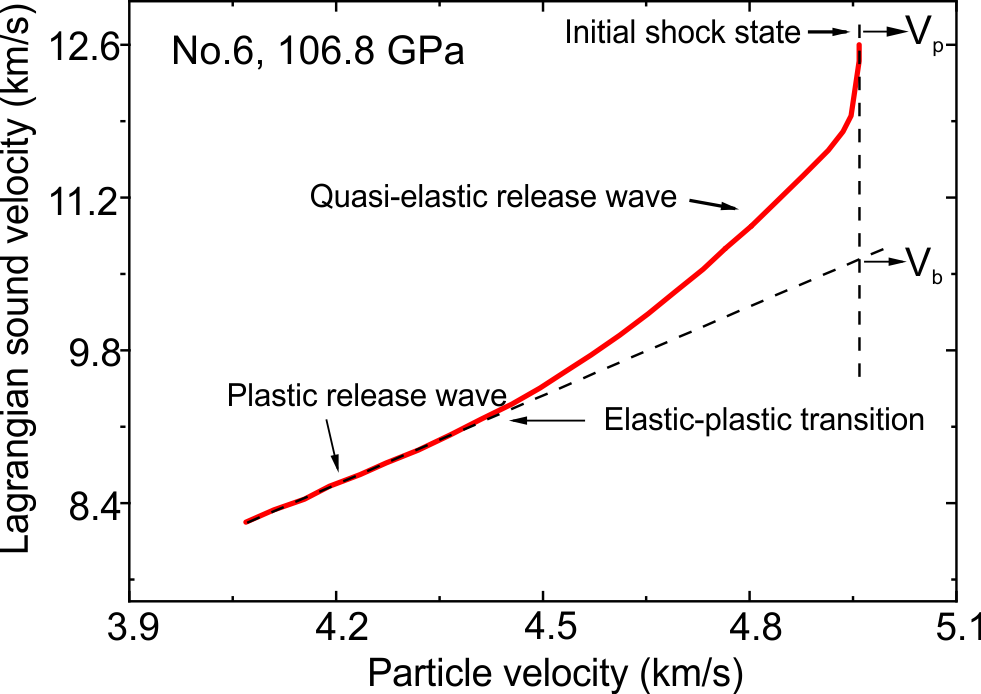


Fig. S2. Typical results for Lagrangian sound velocity versus particle velocity of Fe-1.5wt.%C sample at 106.8 GPa in Shot No. 6. *V_p_* and *V_b_* represent the Lagrangian longitudinal and bulk sound velocity, respectively. The former is directly measured and the latter is deduced by linear extrapolation.


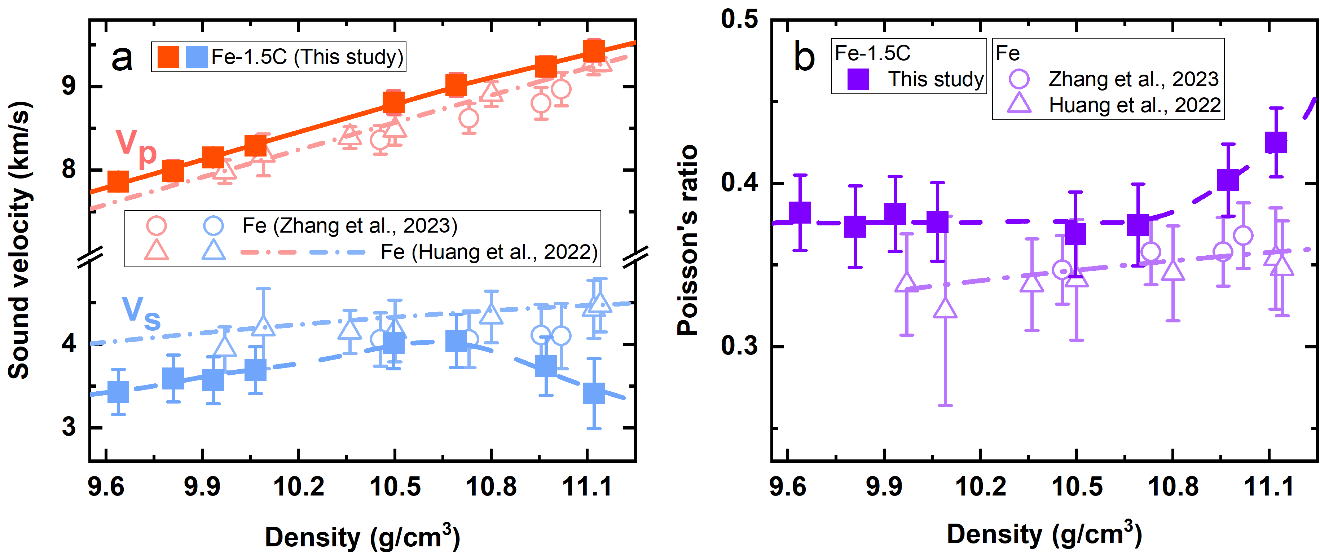


**Fig. S3. Sound velocities and Poisson's ratio of Fe-1.5C and Fe as functions of density under shock compression. a** Sound velocities versus density. The solid squares with error bars represent the experimental results for Fe-1.5C alloy from this study and the red solid lines are the segmented linear fitting to the data, corresponding to the pressure ranges of 49.8-106.8 GPa and 106.8-140.0 GPa, respectively. The bule dashed curve is the result of polynomial fitting to the data. All open symbols with error bars are the literature data for Fe. The circles are from Zhang et al. (2023) [7] and the triangles along with the dash-dot lines are from Huang et al. (2022) [8]. The symbols *V_p_* (in red) and *V_s_* (in blue) indicate the longitudinal and shear sound velocities, respectively. b  Poisson's ratio versus density. The purple squares with error bars are the data for Fe-1.5C alloy from this study, and the purple open circles and triangles with error bars are the data from Zhang et al. (2023) [7] and Huang et al. (2022) [8], respectively. The dashed and dash-dot lines illustrate the variations of Poisson’s ratio with density for Fe-1.5C alloy and pure iron, respectively.


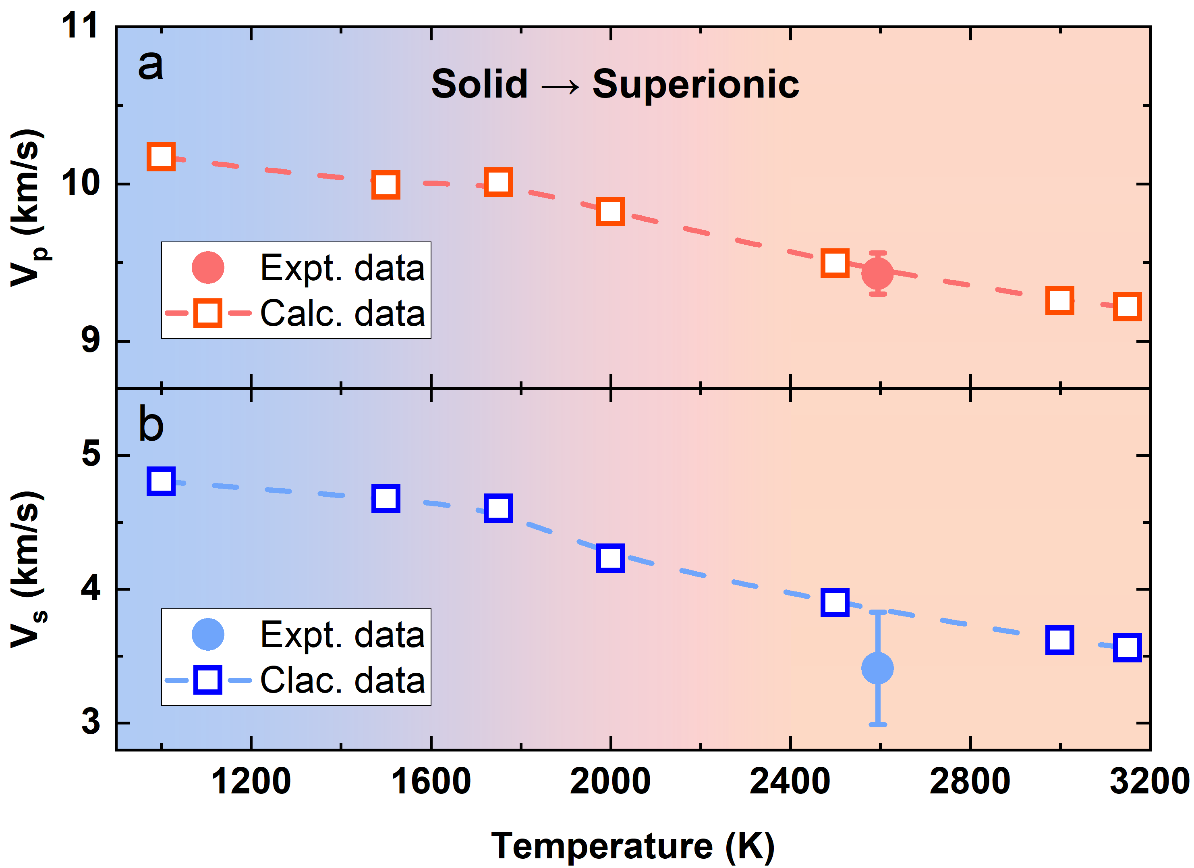


Fig. S4. Sound velocities of Fe-1.5C alloy as a function of temperature at 140 GPa. Red and blue symbols represent data for longitudinal (*V_p_*) and shear (*V_s_*) sound velocities, respectively. The solid symbols are the experimental results at 140 GPa and ~2,600 K, while the open symbols are the calculated data at 140 GPa and various temperatures.


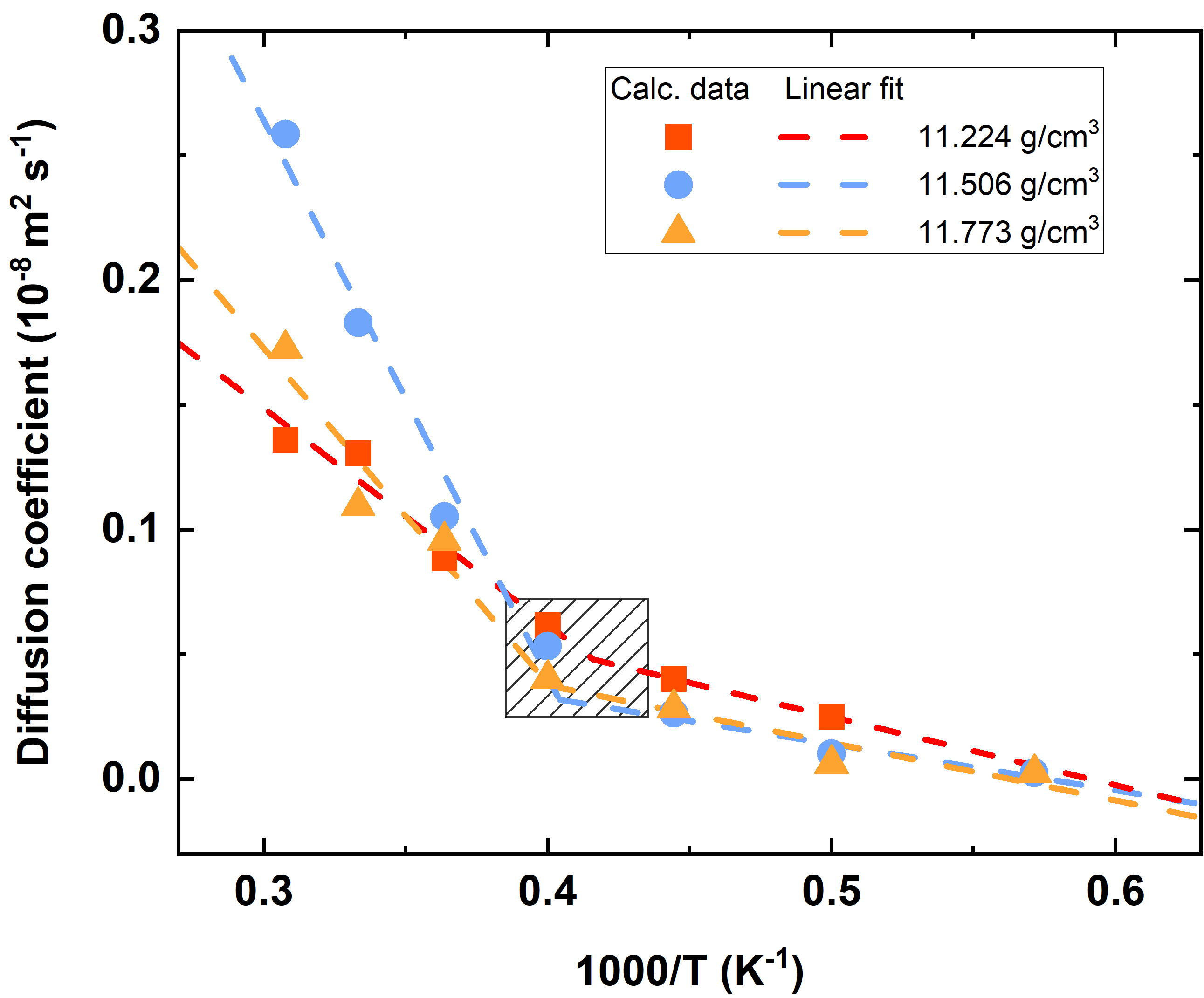


Fig. S5. Diffusion coefficients of carbon atoms in solid-hcp and superionic-hcp iron alloy. Red squares, blue circles, and orange triangles represent estimated diffusion coefficients of carbon atoms for Fe-1.5C alloy at 11.224, 11.506, and 11.773 g/cm^3^, respectively. The dashed lines are the linear fits to the corresponding calculated data. The hatch rectangle highlights the regions of significant change in diffusion coefficients, corresponding to solid-superionic phase transition of Fe-1.5C alloy.


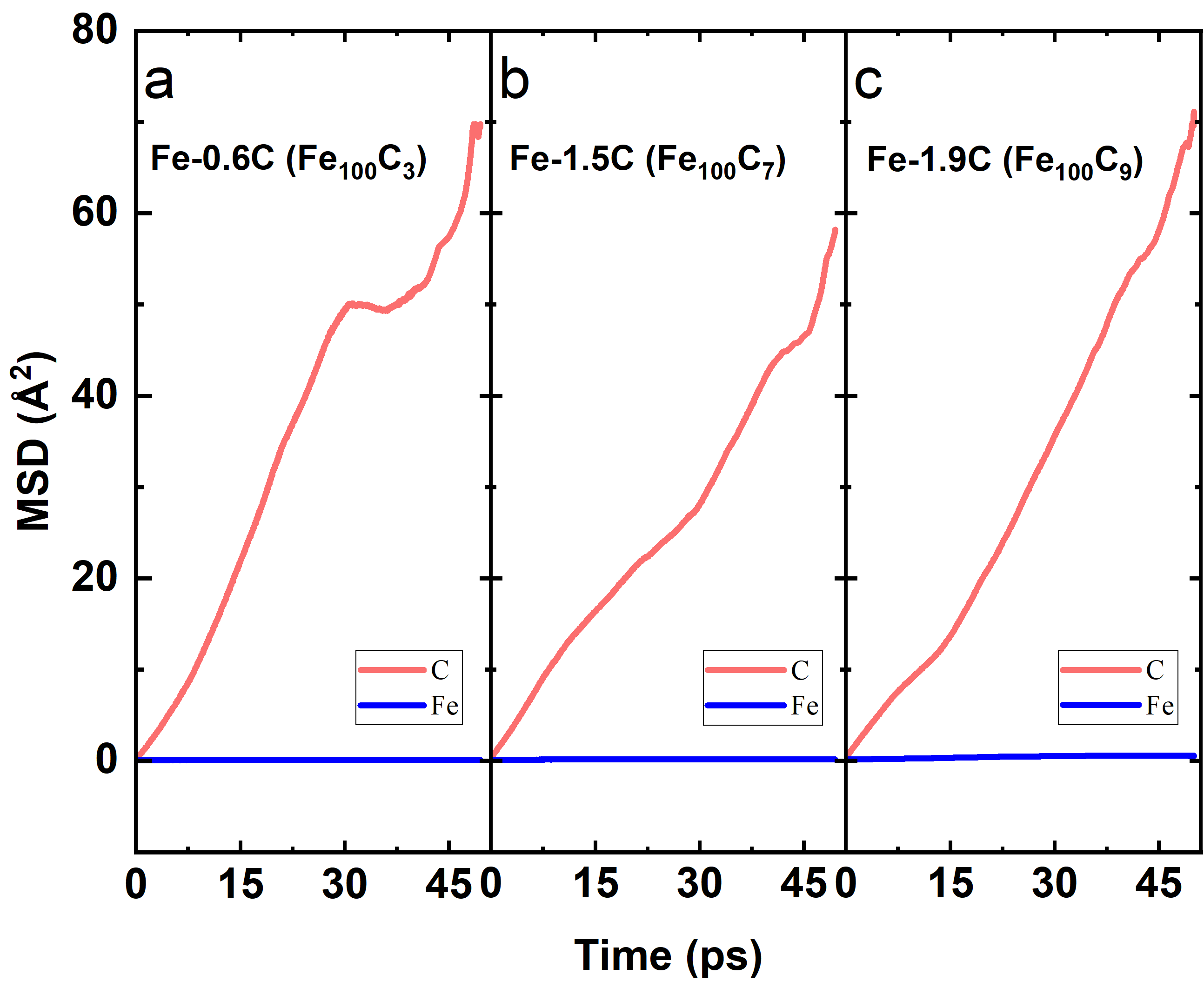


Fig. S6. Calculated MSDs of carbon and iron atoms in Fe-0.6C (Fe_100_C_3_), Fe-1.5C (Fe_100_C_7_), and Fe-1.9C (Fe_100_C_9_) at ~166 GPa and 3000 K. Orange and blue curves represent MSDs of carbon and iron atoms, respectively. The volume of supercells keeps constant to be 818.09 Å^3^. In all the compositions, the MSDs of iron remain to be nearly zero while those of carbon are estimated to be 0.2 × 10^-8^ m^2^ s^-1^.

**
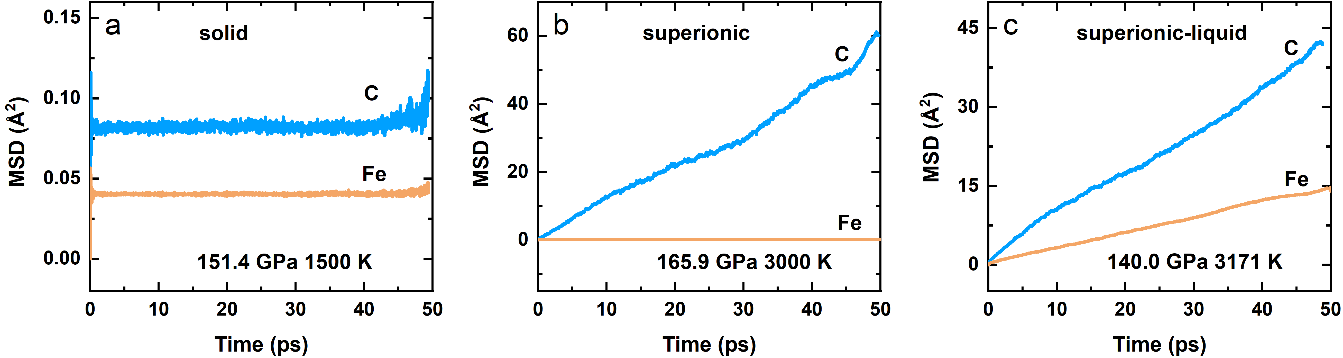
**

**Fig. S7. Calculated MSDs of C and Fe in Fe-1.5C alloy in normal solid, superionic, superionic-liquid coexisting phase, and superheated superionic phase. a** Solid phase at 151.4 GPa and 1500 K, where both C and Fe atoms show negligible diffusion. **b** Superionic phase at 165.9 GPa and 3000 K, with C atoms diffusing significantly while Fe atoms exhibit minimal diffusion. **c** Superionic - liquid coexisting phase at 140.0 GPa and 3171 K, where both C and Fe atoms display notable diffusion.


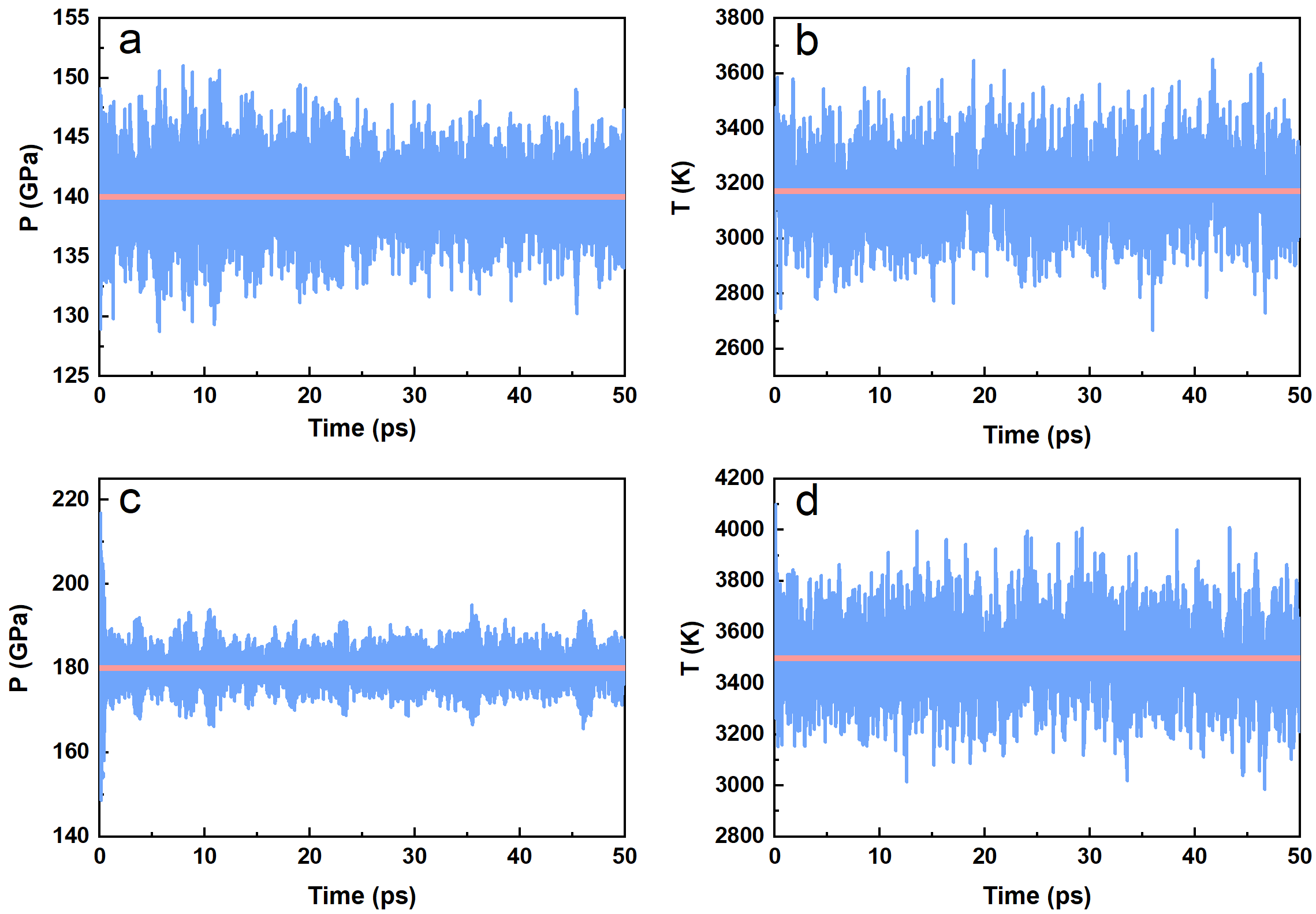


Fig. S8. The fluctuations of pressure and temperature over the simulation time in the two-phase coexisting system for Fe-C alloy. a, c Pressure and b, d temperature for Fe-1.5C alloy are shown in blue curves, and the averaged data are shown with red lines.


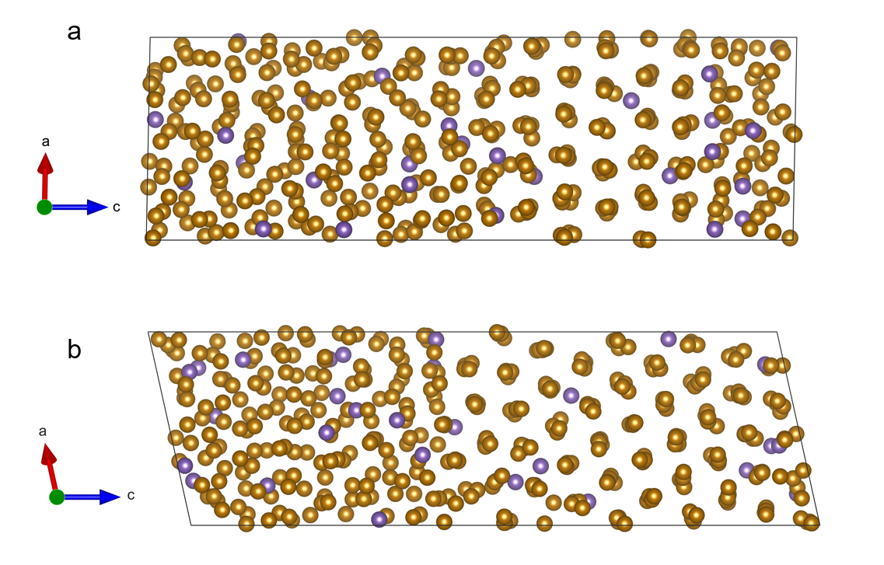


**Fig. S9. Atomic configurations in two-phase coexisting states of Fe-1.5C system after 50 ps in ab initio molecular dynamics simulations.** Two phases coexist at **a** 140 GPa and 3,172 K, and **b** 180 GPa and 3,496 K. Iron and carbon atoms are shown with brown and purple spheres.

**Table S1. Experimental Conditions and Measured Eulerian Sound Velocities of Fe-1.5wt.%C Alloy Under Shock Compression**

| Shot No. | W  (km/s) | ρ_0_  (g/cm^3^) | h_s_  (mm) | U_s_  (km/s) | u_p_  (km/s) | P_H_  (GPa) | ρ  (g/cm^3^) | T_H_  (K) | T_H_/T_m_ | V_p_  (km/s) | V_b_  (km/s) | V_s_  (km/s) | ν |
| --- | --- | --- | --- | --- | --- | --- | --- | --- | --- | --- | --- | --- | --- |
| 1 | 3.406 (7) | 7.768 (10) | 1.688 (2) | 5.746 (49) | 1.115 (5) | 49.8 (3) | 9.638 (26) | 729 (35) | 0.30 | 7.86 (13) | 6.79 (14) | 3.43 (30) | 0.382 (26) |
| 2 | 3.708 (7) | 7.793 (10) | 1.313 (1) | 5.907 (55) | 1.214 (6) | 55.9 (4) | 9.810 (30) | 839 (48) | 0.33 | 7.99 (14) | 6.83 (14) | 3.59 (30) | 0.373 (27) |
| 3 | 4.024 (8) | 7.774 (10) | 1.656 (1) | 6.083 (54) | 1.323 (6) | 62.5 (4) | 9.934 (30) | 932 (59) | 0.36 | 8.15 (14) | 7.03 (14) | 3.57 (32) | 0.381 (27) |
| 4 | 4.366 (9) | 7.755 (10) | 1.716 (2) | 6.273 (52) | 1.440 (6) | 70.0 (5) | 10.066 (31) | 1044 (74) | 0.39 | 8.29 (13) | 7.11 (14) | 3.69 (30) | 0.376 (26) |
| 5 | 5.357 (11) | 7.762 (10) | 1.279 (1) | 6.816 (62) | 1.775 (8) | 93.9 (6) | 10.496 (40) | 1514 (145) | 0.53 | 8.81 (17) | 7.49 (15) | 4.02 (35) | 0.369 (30) |
| 6 | 5.846 (12) | 7.761 (10) | 1.731 (2) | 7.086 (66) | 1.942 (9) | 106.8 (8) | 10.691 (44) | 1788 (191) | 0.61 | 9.02 (17) | 7.72 (15) | 4.04 (36) | 0.375 (29) |
| 7 | 6.478 (7) | 7.792 (10) | 1.656 (2) | 7.428 (37) | 2.153 (5) | 124.6 (7) | 10.972 (28) | 2208 (268) | 0.72 | 9.24 (12) | 8.17 (16) | 3.74 (35) | 0.402 (22) |
| 8 | 7.002 (9) | 7.758 (10) | 1.648 (1) | 7.724 (43) | 2.336 (6) | 140.0 (8) | 11.121 (33) | 2594 (346) | 0.83 | 9.43 (13) | 8.57 (17) | 3.41 (42) | 0.425 (21) |

Note: The first six experiments were conducted at Sichuan University, while the last two experiments were carried out at the China Academy of Engineering Physics. *W* refers to the projectile velocity; *ρ_0_* and *h_s_* denote the initial density and thickness of experimental samples, respectively; the shock wave velocity (*U_s_*), particle velocity (*u_p_*) and Hugoniot pressure (*P_H_*) of Fe-1.5wt.%C sample were determined from the measured *W* and the established Hugoniot equation of state for both sample and LiF window; *ρ* represents the density of sample along Hugoniot; *T_H_* refers to the estimated temperature derived from thermodynamic calculations ($dT=-T\left( \frac{\gamma}{V} \right)dV+\left( \frac{1}{2C_{V}} \right)\left[ \left( V_{0}-V \right)dP+\left( P-P_{0} \right)dV \right]$) utilizing empirical Hugoniot parameters [1,9]. *T_m_* refers to the melting temperature derived from theoretical models, as detailed in Figure 3. Shear wave velocity (*V_s_*) can be deduced from the measured longitudinal (*V_p_*) and bulk velocity (*V_b_*) of Fe-1.5wt.%C alloy under Hugoniot conditions. Subsequently, the Poisson’s ratio (*ν*) is ascertained. The Hugoniot equation of state adopted in this study as following: Fe-1.5wt.%C alloy, *ρ_0_* = 7.771 g/cm^3^, *U_s_* = 1.62 *u_p_* + 3.94 [1]; LiF, *ρ_0_* = 2.638(2) g/cm^3^, *U_s_* = 1.323(9) *u_p_* + 5.201(25) [10]. The standard deviations are given in parentheses estimated through the uncertainty propagation [7].

**Supplementary reference**

S1. Huang Y, Hou M, Gan B *et al.* Iron-Carbon Alloy Under Shock Compression: Implications for the Carbon Concentration in Earth’s Inner Core. *J Geophys Res Solid Earth* 2022;**127**:e2021JB023645.

S2. Hirose K, Wood B, Vočadlo L. Light elements in the Earth’s core. *Nat Rev Earth Environ* 2021;**2**:645–58.

S3. He Y, Sun S, Kim DY *et al.* Superionic iron alloys and their seismic velocities in Earth’s inner core. *Nature* 2022;**602**:258–62.

S4. Sun S, He Y, Yang J *et al.* Superionic effect and anisotropic texture in Earth’s inner core driven by geomagnetic field. *Nat Commun* 2023;**14**:1656.

S5. Vočadlo L. Ab initio calculations of the elasticity of iron and iron alloys at inner core conditions: Evidence for a partially molten inner core? *Earth Planet Sci Lett* 2007;**254**:227–32.

S6. Vočadlo L, Alfè D, Gillan MJ *et al.* The properties of iron under core conditions from first principles calculations. *Phys Earth Planet Inter* 2003;**140**:101–25.

S7. Zhang Y, Wang Y, Huang Y *et al.* Collective motion in hcp-Fe at Earth’s inner core conditions. *Proc Natl Acad Sci USA* 2023;**120**:e2309952120.

S8. Huang H, Fan L, Liu X *et al.* Inner core composition paradox revealed by sound velocities of Fe and Fe-Si alloy. *Nat Commun* 2022;**13**:616.

S9. Brown JM, McQueen RG. Phase transitions, grüneisen parameter, and elasticity for shocked iron between 77 GPa and 400 GPa. *J Geophys Res* 1986;**91**:7485–94.

S10. Liu Q, Zhou X, Zeng X *et al.* Sound velocity, equation of state, temperature and melting of LiF single crystals under shock compression. *J Appl Phys* 2015;**117**:045901.
